# Interim Recommendation of the Advisory Committee on Immunization Practices for Use of the Novavax COVID-19 Vaccine in Persons Aged ≥18 years — United States, July 2022

**DOI:** 10.15585/mmwr.mm7131a2

**Published:** 2022-08-05

**Authors:** Evelyn Twentyman, Megan Wallace, Lauren E. Roper, Tara C. Anderson, Amy B. Rubis, Katherine E. Fleming-Dutra, Elisha Hall, Joy Hsu, Hannah G. Rosenblum, Monica Godfrey, W. Roodly Archer, Danielle L. Moulia, Laura Daniel, Oliver Brooks, H. Keipp Talbot, Grace M. Lee, Beth P. Bell, Matthew Daley, Sarah Meyer, Sara E. Oliver

**Affiliations:** ^1^National Center for Immunization and Respiratory Diseases, CDC; ^2^National Center for Environmental Health, CDC; ^3^Center for Global Health, CDC; ^4^Watts HealthCare Corporation, Los Angeles, California; ^5^Vanderbilt University School of Medicine, Nashville, Tennessee; ^6^Stanford University School of Medicine, Stanford, California; ^7^University of Washington, Seattle, Washington; ^8^Institute for Health Research, Kaiser Permanente Colorado, Denver, Colorado.

The NVX-CoV2373 (Novavax) COVID-19 vaccine is a recombinant spike (rS) protein nanoparticle vaccine with Matrix-M adjuvant to protect against infection with SARS-CoV-2, the virus that causes COVID-19. On July 13, 2022, the Food and Drug Administration (FDA) issued Emergency Use Authorization (EUA) for the Novavax vaccine for primary COVID-19 immunization of unvaccinated adults aged ≥18 years, administered as 2 doses (5 *μ*g rS and 50 *μ*g Matrix-M adjuvant in each dose) 3 weeks apart (*1*). On July 19, 2022, the Advisory Committee on Immunization Practices (ACIP) issued an interim recommendation for use of the Novavax vaccine in persons aged ≥18 years for the prevention of COVID-19.* In the per-protocol^†^ efficacy analysis, vaccine efficacy (VE) against reverse transcription–polymerase chain reaction (RT-PCR)–confirmed symptomatic COVID-19 was 89.6% (95% CI = 82.4%–93.8%). The Alpha variant (B.1.1.7) of SARS-CoV-2 was the predominant circulating variant during the period of case accrual for VE assessments. Cases of myocarditis or pericarditis were reported in temporal association with vaccination, suggesting a possible causal relationship. The ACIP recommendation for the use of the Novavax COVID-19 vaccine is interim and will be updated as additional information becomes available. The adjuvanted, protein subunit-based Novavax COVID-19 vaccine provides an additional option for unvaccinated adults, increasing flexibility for the public and for vaccine providers. Vaccination is important for protection against COVID-19.

Since June 2020, ACIP has convened 31 public meetings to review data relevant to the epidemiology of COVID-19 and considerations for the use of COVID-19 vaccines, including the Novavax vaccine.^§^ The ACIP COVID-19 Vaccines Work Group (Work Group), comprising experts in adult and pediatric medicine, infectious diseases, vaccinology, vaccine safety, public health, and ethics, has held weekly meetings to review COVID-19 surveillance data, evidence for VE, postauthorization effectiveness, safety, and implementation considerations for COVID-19 vaccines. To guide its deliberations regarding recommendations for use of these vaccines, ACIP used the Evidence to Recommendation (EtR) Framework^¶^ and incorporated a Grading of Recommendations, Assessment, Development, and Evaluation (GRADE) approach** (*2*). Within the EtR Framework, ACIP considered the importance of COVID-19 as a public health problem, as well as population values and preferences, acceptability, feasibility, resource use, and equity regarding use of the Novavax COVID-19 vaccine among persons aged ≥18 years. After conducting systematic reviews of published and unpublished evidence for benefits and harms, the Work Group used the GRADE approach to independently assess the certainty of evidence for outcomes related to the Novavax COVID-19 vaccine, rated on a scale of type 1 (high certainty) to type 4 (very low certainty)^††^ (*2*). Work Group conclusions regarding evidence for use of the Novavax COVID-19 vaccine among persons aged ≥18 years were presented to ACIP at a public meeting on July 19, 2022.

## Summary of Evidence for Use of the Novavax COVID-19 Vaccine in Persons Aged ≥18 Years

The body of evidence regarding efficacy of the Novavax COVID-19 vaccine among persons aged ≥18 years consisted of data from one randomized, double-blind, placebo-controlled phase III clinical trial (2019nCoV-301), based in the United States and Mexico, in which 29,945 participants aged ≥18 years were enrolled and randomized 2:1 to receive 2 intramuscular doses of either Novavax COVID-19 vaccine (5 *μ*g rS and 50 *μ*g Matrix-M adjuvant) or saline placebo, separated by an interval of 3 weeks (*3*). Upon completion of the assigned study arm (i.e., receipt of either 2 doses of vaccine or 2 doses of placebo), trial participants were offered the opportunity to cross over in a blinded fashion (“blinded crossover”) from their originally assigned study arm. Therefore, all trial participants had the opportunity to receive trial vaccine in either the precrossover or postcrossover period, while remaining blinded throughout. Efficacy and safety data are available from the precrossover period (December 27, 2020–September 27, 2021) of this trial, and these data comprise the basis of the assessment made using the GRADE approach. Data from additional sources were considered in assessment of benefits and harms, as guided by the EtR Framework. These sources included additional safety data available from the postcrossover period of 2019nCoV-301, safety data from three additional Novavax clinical trials based in the United Kingdom (2019nCoV-302), South Africa (2019nCoV-501), and Australia (2019nCoV-101), postmarketing safety data submitted to FDA by Novavax summarizing administration of 744,235 doses of Novavax COVID-19 vaccine globally as of April 30, 2022 (*4*), and publicly available data pertaining to the administration of 160,000 Novavax COVID-19 vaccine doses in Australia as of June 26, 2022 (*5*).

The primary efficacy endpoint was diagnosis of RT-PCR–confirmed symptomatic COVID-19 ≥7 days after receipt of the second dose in the 2-dose primary series of Novavax COVID-19 vaccine arm compared with that in the placebo arm. Per the manufacturer’s trial protocol, COVID-19 cases were only included in the efficacy analysis if they were RT-PCR–confirmed by a designated central laboratory. The primary efficacy endpoint was assessed until a participant received the first blinded crossover vaccination or until the data cutoff date of September 27, 2021, whichever occurred earlier. The per-protocol VE analysis population included 17,272 Novavax COVID-19 vaccine recipients and 8,385 placebo recipients with a median 2.5 months of blinded follow-up after receipt of dose 2. VE^§§^ against RT-PCR–confirmed symptomatic COVID-19, observed during the period of Alpha variant predominance, was 89.6% (95% CI = 82.4%–93.8%) in persons aged ≥18 years without evidence of previous SARS-CoV-2 infection.^¶¶^ This estimate was based on symptomatic illness in 17 vaccine recipients and 79 placebo recipients, none of whom was hospitalized. VE against severe COVID-19*** was 100%, based on four severe cases in the placebo group and none in the vaccine group. Subgroup analyses by age demonstrated VE against RT-PCR–confirmed symptomatic COVID-19 of 90.3% (95% CI = 83.1%–94.4%) among participants aged 18–64 years and 76.3% (95% CI = 29.1%–95.7%) among participants aged ≥65 years. A post hoc analysis of VE^†††^ among participants aged 50–64 years demonstrated VE of 90.7% (95% CI = 72.9%–96.8%) against RT-PCR–confirmed symptomatic COVID-19. Additional evidence pertaining to potential VE in persons aged ≥65 years was provided through immunobridging. The measure of immune response to 2 doses of Novavax COVID-19 vaccine in persons aged ≥65 years without evidence of previous SARS-CoV-2 infection was slightly lower than that observed in persons aged 50–64 years, with a geometric mean ratio (GMR) for day 35 neutralizing antibody titer of 0.91 (95% CI = 0.68–1.2) for persons aged ≥65 years, demonstrating estimates that would have met FDA’s usual success criterion for immunobridging noninferiority.^§§§^ Subgroup analyses of VE against RT-PCR–confirmed symptomatic COVID-19 by ethnicity and race demonstrated that most estimates by ethnicity and race (when calculable from the data) were comparable to the per-protocol VE overall, but VE in participants of Hispanic or Latino (Hispanic) ethnicity was lower (75.7%; 95% CI = 46.0%–89.1%).

Among vaccine recipients aged ≥18 years, reactogenicity, defined as solicited local and systemic reactions during the 7 days after vaccination, was reported frequently. Following dose 2, 78.6% of participants reported any local reactions, and 69.3% reported any systemic reactions. Most solicited reactions were mild to moderate and lasted 1–3 days; all were reported more frequently after dose 2 than after dose 1 among vaccine recipients. Severe solicited reactions (grade 3 or higher, defined as interfering with daily activity) were more common in vaccine (16.3%) than placebo (4.0%) recipients. Severe solicited local reactions occurred in 1.1% of vaccine recipients after dose 1 and in 6.7% after dose 2, compared with 0.2% and 0.3%, respectively, in placebo recipients. Severe solicited systemic reactions occurred in 2.4% of vaccine recipients after dose 1 and in 12.1% after dose 2, compared with 2.1% after each dose among placebo recipients. Reported reactions varied with age and were more common among vaccine recipients aged 18–64 years than among those aged ≥65 years. Among vaccine recipients aged 18–64 years, the most common reactions associated with any vaccine dose included injection site pain or tenderness (82.2%), fatigue (62.0%), muscle pain (54.1%), and headache (52.9%). In participants aged ≥65 years, the most common vaccine-associated reactions associated with any dose included injection site pain or tenderness (63.4%), fatigue (39.2%), muscle pain (30.2%), and headache (29.2%). The most common grade 3 or higher local reaction reported by vaccine recipients after dose 2 was pain or tenderness at the injection site (6.3%). The most common grade 3 or higher systemic reaction reported by vaccine recipients after dose 2 was fatigue (10.5%). Reports of unsolicited serious adverse events^¶¶¶^ were comparable between vaccine (1.0%) and placebo (1.1%) recipients in the precrossover period and among participants who crossed over to receive Novavax COVID-19 vaccine (1.4%) and placebo (1.2%) in the postcrossover period.

Cases of myocarditis or pericarditis were detected in Novavax clinical trials. Among a total of 41,546 vaccine recipients aged ≥16 years, including within both precrossover and postcrossover vaccine arms in 2019nCoV-301, as well as all vaccine recipients in 2019nCoV-302, 2019nCoV-501, and 2019-nCoV101 combined, six cases of myocarditis or pericarditis were detected; five occurred within 20 days of vaccination. Among these five, four did not have likely alternative etiologies, suggesting a possible causal relationship with vaccine. Cases of myocarditis or pericarditis have also been detected in global postauthorization surveillance; during a period in which 744,235 doses of Novavax COVID-19 vaccine were administered in Australia, Canada, the European Union, New Zealand, and South Korea, 35 reports (representing 36 adverse events) were identified among 20 male and 15 female vaccine recipients with a median age of 34 years (range = 23–62 years): 29 reports of pericarditis, including five in persons with a history of pericarditis after mRNA COVID-19 vaccine; four myocarditis cases; two myopericarditis cases; and one case of carditis, not otherwise specified. A postmarketing analysis from Australia identified three cases of myocarditis and 12 cases of pericarditis reported during a period in which 160,000 Novavax COVID-19 vaccine doses were administered (*5*).

In addition to the myocarditis or pericarditis cases detected in Novavax clinical trials, one case of angioedema (2019nCoV-301) and one case of Guillain-Barré syndrome (2019nCoV-302) were also considered to be potentially related to vaccination. A detailed summary of safety data, including information on reactogenicity, is available at https://www.cdc.gov/vaccines/covid-19/info-by-product/novavax/reactogenicity.html.

From GRADE evidence assessment, the level of certainty for the benefits of Novavax COVID-19 vaccination among persons aged ≥18 years was type 1 (high certainty) for the prevention of symptomatic laboratory-confirmed COVID-19 assessed using direct efficacy. Evidence was type 3 (low certainty) for prevention of COVID-19–associated hospitalization. No COVID-19–associated hospitalizations were present in the per-protocol trial efficacy analysis; therefore, the outcome was evaluated using severe COVID-19 as a surrogate measure. Concerns of imprecision arose because of the small number of severe COVID-19 events. Regarding potential harms after vaccination, evidence was type 1 (high certainty) for serious adverse events and reactogenicity of grade 3 or higher. No data were available to assess other GRADE benefits, including prevention of death due to COVID-19 or prevention of asymptomatic SARS-CoV-2 infection.

## Recommendations for Use of the Novavax COVID-19 Vaccine in Persons Aged ≥18 Years

Data reviewed within the EtR Framework supported use of the Novavax COVID-19 vaccine in persons aged ≥18 years. COVID-19 remains a major public health problem in the United States. As of July 14, 2022, approximately 89 million COVID-19 cases have occurred in the United States, and outcomes have been most severe among adults: of 4,936,004 COVID-19–related hospitalizations in the United States during August 1, 2020–July 10, 2022, most (4,798,764; 97.2%) were among persons aged ≥18 years; and >99% of 1,015,431 COVID-19–related deaths in the United States through July 13, 2022, were among persons aged ≥18 years (*6*,*7*). COVID-19 continues to expose longstanding health inequities in the United States, as racial and ethnic minority populations continue to experience a disproportionate incidence of COVID-19 (*8*).

The benefits of receiving a primary COVID-19 vaccination series with authorized or approved vaccines are clear (*1*,*3*,*9*). Outcomes of COVID-19 among persons who are not vaccinated against COVID-19 are significantly worse than those among persons who have received at least a primary vaccination series; in April 2022, among persons aged ≥5 years, unvaccinated persons had six times the risk of dying from COVID-19 compared with those who had received a primary COVID-19 vaccination series (*9*). However, the benefits of Novavax COVID-19 vaccine in the context of currently circulating SARS-CoV-2 variants is unknown because VE was assessed during the period of Alpha predominance. Direct evidence of efficacy against the Omicron variant or any of its sublineages is not yet available; evaluation of Novavax COVID-19 VE, including the subgroups in which VE in the trial appeared to have been potentially relatively lower (persons aged ≥65 years and persons of Hispanic ethnicity), will be based on postauthorization surveillance.

In evaluation of potential harms, cases of myocarditis or pericarditis were detected during Novavax COVID-19 vaccine clinical trials; in addition, reports of myocarditis or pericarditis were identified during postauthorization use of Novavax COVID-19 vaccine in countries outside the United States (*1*). These reports underscore the critical importance of ongoing vaccine safety surveillance in the United States and internationally to help assess the possible risk for Novavax COVID-19 vaccine-associated myocarditis or pericarditis, in addition to ongoing monitoring of myocarditis or pericarditis after receipt of mRNA COVID-19 vaccines and continued communication with health care providers.

As of May 2022, an estimated 13.9% of persons aged ≥18 years in the United States remained unvaccinated against COVID-19 (*10*). The availability of a COVID-19 vaccine that uses more traditional vaccine technology provides an additional option for unvaccinated persons. Recent data reflect some interest in protein-based adjuvanted vaccines such as the Novavax COVID-19 vaccine (*2*). A survey designed to assess vaccination intentions for adjuvanted or nonadjuvanted protein-based COVID-19 vaccines among unvaccinated Americans conducted during January–February 2022 found that 16.0% of unvaccinated adults said that they “probably” or “definitely” would get an adjuvanted protein-based vaccine, with significantly lower intent among non-Hispanic White persons (9.6%) than among non-Hispanic Black or African American (20.1%) and Hispanic persons (19.5%), and with significantly higher intent among men (21.8%) than among women (11.9%) (*2*).

Although uncertainty remains in the valuation of the vaccine by all populations, it was determined that for most populations, the desirable effects of Novavax COVID-19 vaccine outweigh any potential undesirable effects. The Novavax COVID-19 vaccine is likely acceptable, and implementation of vaccination feasible, particularly in the context of knowledge gained over the course of >1.5 years of distribution and administration of COVID-19 vaccines.

Before vaccination, the EUA Fact Sheet should be provided to vaccine recipients and their caregivers. Novavax COVID-19 vaccine is authorized as a 2-dose primary series separated by 3 weeks. Some studies with mRNA COVID-19 vaccines (*11*) have indicated that the small risk of myocarditis or pericarditis might be reduced with a longer interval between doses; however, no data currently exist for Novavax COVID-19 vaccine. Consequently, based on evidence of benefits of an extended interval in persons receiving mRNA COVID-19 vaccines, an 8-week interval between Novavax doses may be selected to potentially reduce the risk for myocarditis or pericarditis after vaccination. Development of myocarditis or pericarditis after a dose of an mRNA COVID-19 vaccine or Novavax COVID-19 vaccine is a precaution to a subsequent dose of any COVID-19 vaccine. Additional clinical guidance is available at https://www.cdc.gov/vaccines/covid-19/clinical-considerations/interim-considerations-us.html. ACIP will continue to review additional data as they become available.

## Reporting of Vaccine Adverse Events

Adverse events that occur after receipt of any COVID-19 vaccine should be reported to the Vaccine Adverse Event Reporting System (VAERS). Vaccination providers are required by FDA to report vaccine administration errors, serious adverse events, cases of multisystem inflammatory syndrome, and cases of COVID-19 that result in hospitalization or death after administration of COVID-19 vaccine under EUA. Reporting is encouraged for any clinically significant adverse event even if it is uncertain whether the vaccine caused the event. Information on how to submit a report to VAERS is available at https://vaers.hhs.gov/index.html or 1-800-822-7967. In addition, CDC has established v-safe, a voluntary smartphone-based active surveillance system that monitors adverse events occurring after COVID-19 vaccination. Reports to v-safe indicating a medically significant health impact are followed up by CDC’s v-safe call center to collect additional information, and complete a VAERS report, if indicated. Information on v-safe is available at https://www.cdc.gov/vsafe.

**Summary**

**What is already known about this topic?**

On July 13, 2022, the Food and Drug Administration issued Emergency Use Authorization for the NVX-CoV2373 (Novavax) COVID-19 vaccine.

**What is added by this report?**

On July 19, 2022, the Advisory Committee on Immunization Practices made an interim recommendation for use of the Novavax vaccine in persons aged ≥18 years as a primary 2-dose series vaccination for the prevention of COVID-19.

**What are the implications for public health practice?**

The adjuvanted, protein subunit–based Novavax COVID-19 vaccine provides an additional option for unvaccinated adults, increasing flexibility for the public and for vaccine providers. Vaccination is important for protection against COVID-19.

## Footnotes

* On July 19, 2022, ACIP voted 12 to 0 (three members absent) in favor of the interim recommendation for use of the Novavax COVID-19 vaccine for persons ≥18 years.

† The per-protocol efficacy analysis population was defined as participants who were randomized, received both doses as assigned, had no evidence of SARS-CoV-2 infection before dose 1, and did not have a COVID-19 event at any time before 7 days after dose 2.

§
https://www.cdc.gov/vaccines/acip/meetings/index.html

¶
https://www.cdc.gov/vaccines/acip/recs/grade/covid-19-novavax-etr.html

**
https://www.cdc.gov/vaccines/acip/recs/grade/covid-19-novavax.html

††
https://www.cdc.gov/vaccines/acip/recs/grade

§§ For GRADE evidence assessment, relative risks (RRs) were calculated from numerators and denominators available in the body of evidence. VE estimates were defined as 100% x (1 – RR). Manufacturer VE estimates were calculated from a log-linear model using modified Poisson regression.

¶¶ In 2019nCoV-301, 6.5% of vaccine recipients and 8.4% of placebo recipients in the precrossover period had evidence of previous infection with SARS-CoV-2 at baseline, as assessed by serology before vaccination.

*** Because no COVID-19–associated hospitalizations occurred in the per-protocol trial efficacy analysis, severe COVID-19 was used as a surrogate measure for COVID-19–associated hospitalization in GRADE assessments. Severe COVID-19 was defined as a COVID-19 case associated with one or more of the following conditions: tachypnea (≥30 breaths per minute at rest), tachycardia (resting heart rate ≥125 beats per minute), hypoxemia (oxygen saturation ≤93% on room air or ratio of the partial pressure of arterial oxygen to the fraction of inspired oxygen <300 mmHg), high-flow oxygen therapy or noninvasive ventilation/noninvasive positive pressure ventilation, mechanical ventilation or extracorporeal membrane oxygenation, or one or more major organ system dysfunction or failure, including any of the following conditions: acute respiratory distress syndrome, acute renal failure, acute hepatic failure, acute right or left heart failure, septic or cardiogenic shock (systolic blood pressure <90 mmHg or diastolic blood pressure <60 mmHg), acute stroke (ischemic or hemorrhagic), acute thrombotic event, requirement for vasopressors, systemic corticosteroids, hemodialysis, admission to an intensive care unit, or death.

††† Post hoc VE was based on manufacturer VE estimate.

§§§ Noninferiority is declared if the lower bound of the two-sided 95% CI for the GMR is >0.67, and the point estimate of GMR is ≥0.8.

¶¶¶ Serious adverse events are defined as death, life-threatening event, inpatient hospitalization or prolongation of existing hospitalization, persistent or significant incapacity or substantial disruption of the ability to conduct normal life functions, important medical event, or congenital anomaly or birth defect. Five participants in the vaccine arm experienced serious adverse events that the investigator considered to be related to vaccination. Among these, FDA considered one event of angioedema as being potentially related to vaccination.
